# Yield of next-generation sequencing in diagnostic work up of suspicious biliary strictures

**DOI:** 10.1055/a-2687-3552

**Published:** 2025-09-05

**Authors:** Tina L. N. Meijering, David M. de Jong, Swip Draijer, Marco J. Bruno, Hendrikus J. Dubbink, Jeroen de Jonge, Marie-Louise F. van Velthuysen, Lydi M. J. W. van Driel

**Affiliations:** 1 Gastroenterology and Hepatology, Erasmus MC University Medical Center Rotterdam, Rotterdam, Netherlands; 2 Pathology, Erasmus MC University Medical Center Rotterdam, Rotterdam, Netherlands; 3 Surgery, Erasmus MC University Medical Center Rotterdam, Rotterdam, Netherlands

**Keywords:** Pancreatobiliary (ERCP/PTCD), Strictures, Tissue diagnosis, Diagnostic ERC, GI Pathology

## Abstract

**Background and study aims:**

This study addressed the need for improved diagnostic tools to identify malignancy in suspicious biliary strictures. Traditional cytological morphology is often indecisive, prompting exploration of next-generation sequencing (NGS) for enhanced sensitivity. Our aim was to evaluate NGS's additional value in classifying biliary brushes and biopsies and its impact on clinical decision making (CDM).

**Patients and methods:**

In this retrospective single-center cohort study, patients were included from 2019 to 2022 in whom morphologic interpretation and NGS were performed on cytological or histological material from suspicious biliary strictures. Sensitivity and specificity of NGS were calculated for benign or atypical vs. suspicious for malignancy or malignant morphology in biliary brushes and biopsies. In addition, changes in CDM after NGS outcome were evaluated.

**Results:**

In total 109 samples from 106 patients were included in the study. NGS correctly identified 42 of 75 malignancies (56%). Sensitivity and specificity of morphology for brushes were 56% (95% confidence interval [CI] 43%-68%) and 94% (95% CI 79%-99%), respectively. Adding NGS resulted in sensitivity and specificity of 78% (95% CI 66%-87%) and 94% (95% CI 79%-99%). For biopsies, sensitivity and specificity of morphology were 67% (95% CI 35%-90%) and 67% (95% CI 9%-99%) and adding NGS did not alter these results. The outcome of NGS resulted in a change of classification of morphology in 36% and a change in CDM in 8%.

**Conclusions:**

NGS in brushes contributed to more accurate/sensitive diagnoses of malignancy than morphology alone. There was a limited impact on CDM change, but in the future, NGS will undoubtedly play a bigger role when targeted therapy is incorporated in standard treatment and more sensitive NGS panels for cholangiocarcinoma are developed.

## Introduction

Malignant biliary obstructions (MBOs) primarily result from cholangiocarcinoma (CCA), gallbladder carcinoma, and pancreatic adenocarcinoma, carrying a very poor prognosis 1 2 . A definite pathological diagnosis is preferred for surgery but warranted before commencing systemic therapy. Approximately 13% of patients undergoing extensive surgery for suspected biliary malignancy have benign pathology in resection specimens 3 4 5 . Unfortunately, an accurate diagnosis is often difficult due to several factors 2 6 .

Firstly, it is difficult to obtain enough tissue inherent to the nature of the tumor. Either endoscopically or percutaneously, tissue can be obtained by bile duct brushes or intraductal biopsies. It is often challenging to confirm malignancy due to sample failure, low cellular yield, or difficulty identifying minimal malignant changes. For example, in cases in which tumor progression is predominately submucosal or periductal, superficial samples may not contain malignant cells or sufficient tumor DNA 2 6 7 . This results in a low mean sensitivity of biliary duct brushes to detect true malignancy of 45%, but a specificity of 99% in case of confirmative pathology 8 . Studies show a high positive predictive value (PPV) ranging from 83% to 99%, and a low negative predictive value (NPV) ranging from 49% to 58% 9 10 11 . Second, atypical cells are difficult to classify because they can either be malignant or benign in case of inflammation. Additional diagnostics to classify these atypical cells often lead to higher costs, delays in treatment, and ensuing uncertainty for the patient 12 13 . The proposed algorithm for diagnosis of bile duct strictures according to the European Society of Gastrointestinal Endoscopy guideline recommends cholangioscopy-guided biopsy after inconclusive endoscopic retrograde cholangiopancreatography (ERCP) with brushes and fluoroscopy-guided biopsy or endoscopic ultrasound-guided tissue acquisition 14 However, recent literature affirms suboptimal sensitivity of 66% for cholangioscopy-guided biopsies 15 . Other additional diagnostics, such as fluorescence in situ hybridization and tumor markers, such as CA-19.9, are suboptimal with sensitivity ranging from 34% to 72% 16 17 18 19 20 . Therefore, a more sensitive diagnostic technique for suspicious biliary strictures is desirable, especially for correct classification of atypical cells.

Next-generation sequencing (NGS) emerged as a promising diagnostic technique for suspicious biliary strictures, enabling sensitive mutation detection in low-abundant tumor DNA 21 . NGS gene panels can identify various oncogene and tumor suppressor mutations involved in bile duct malignancies, including targetable alterations such as *ERBB2* 22 23 . Preliminary studies show that NGS on brushes and biopsies increases sensitivity significantly to 77% to 93%, while maintaining a high specificity of 99% to 100%. However, its impact on clinical decision-making (CDM) remains unclear 23 24 25 . Furthermore, there is limited knowledge about the group of patients with morphology classified as atypical and subsequent NGS.

The aim of this study was to assess the ability of NGS to correctly diagnose MBOs from biliary brush and biopsy samples, particularly in patients with indecisive morphology and to determine the effect of NGS on CDM.

## Methods

### Study design and population

A retrospective, single-center cohort study was performed at Erasmus MC University Medical Center, a Dutch tertiary referral center for pancreato-biliary diseases. The eligible study population consisted of all patients with biliary obstructions from whom specimens were obtained by brush or biopsy and in whom NGS subsequently was performed. Patients who received chemotherapy or surgery prior to the brush or biopsy were excluded. Since January 2019 NGS, has been performed on request of the clinician, especially in cases with indecisive morphologic cytology diagnosis. There was no agreed-upon clinical indication for when to perform NGS; the clinician determined the indication for NGS. Eligible patients were retrospectively identified from our local database, the Decentral PALGA system (DPS), between January 2019 and May 2022. The study followed the Helsinki Declaration guidelines and the local ethics committee waived the need for informed consent due to its retrospective nature (MEC-2022–0403).

### Brushes and biopsy procedure

Suspicious biliary strictures were sampled using brushes or biopsies during standard ERCP or percutaneous transhepatic biliary drainage (PTBD). At least two consecutive brushes were performed followed by intraductal biopsies if possible. During the brush procedure, multiple (usually about 10) back-and-forth motions were made at the desired location. Subsequently, the brushes were put together in CytoLyt preservative solution and sent for cytological analysis. In selected cases, cholangioscopy-guided biopsies were performed. The outcome of the analysis was classified as benign, atypical, suspicious for malignancy, or malignant by highly experienced pathologists with > 10 years expertise in both cytology and histology. For referred patients, the original material from brushes or biopsies was reassessed and used for NGS.

## Next-generation sequencing

DNA was isolated from biopsy or brush material 26 . Depending on the NGS panel, a minimum of 1 or 10 ng DNA inputs was used for NGS analysis using the Ion Torrent sequencing system (Thermo Fisher Scientific, United States) to identify insertions/deletions, point mutations, and copy number alterations (amplifications or deletions). Three different NGS panels, detailed in **Supplementary Text 1** , were utilized for analysis. Depending on the material quantity and estimated tumor component, custom-made pan-cancer diagnostic panels or the ThermoFisher Oncomine Colon cell-free DNA (cfDNA) Assay V1 was utilized. The pan-cancer diagnostic panel was used as the standard panel because of the wide range of genes that can be examined simultaneously. Analyses before January 2020 used version 5.1 and examined 41 genes 27 . After January 2020, version 6.1 was used, analyzing 19 additional genes. These pan-cancer panels required a minimum of 10 ng DNA, ≥ 10% variant allele frequency, and ≥ 20% neoplastic cells for reliable detection of mutations and copy number variations. Allele frequency was reported as a percentage of the variant allele frequency in proportion to the wild type allele. For samples with ≤ 10% tumor cells or uncertain tumor content, the cfDNA panel analyzed the 14 most commonly mutated genes in gastrointestinal carcinomas, although it did not assess copy number variations. The cfDNA assay required one to 50 ng DNA and the limit of detection was calculated by the data analysis pipeline of the Torrent Suite (Thermo Fisher Scientific) and varied from 0.09% to 4.88%. In some cases, the initial NGS analysis was extended with an additional panel (cfDNA or pan-cancer) to detect possible missed mutations.

A positive NGS result was defined by presence of one or more pathogenic mutations in known oncogenes or suppressor genes. Most common mutations in CCA are *KRAS* , *TP53* , *SMAD4* , *CDKN2A,* or *BRAF* 23 25 . Single mutations in *KRAS* or *GNAS* , variants of unknown significance, or mutations with a very low, unreliable allele frequency (< 1%) were labelled “not sufficient for malignancy” and, therefore, classified as a negative NGS result. A negative result was also given if no mutations were found or NGS was unsuccessful.

### Primary outcome

The primary outcome of this study was to evaluate the sensitivity, specificity, accuracy, PPV, and NPV of NGS in suspicious biliary strictures. Final diagnosis of the suspicious biliary strictures was based on surgical resection specimens, autopsy, other biopsies, and/or clinical follow-up. Malignancy was defined as disease progression on imaging, clinical progression, or death due to clinically or radiologically determined MBO. Benign pathology was determined with resolution or no lesion progression on imaging during ≥ 6 months of follow-up. In calculations for morphology, benign/atypical morphology are considered a negative test outcome, whereas suspicious for malignancy/malignant morphology are considered a positive test outcome.

### Secondary outcome

The secondary outcome was CDM changes due to NGS. A select group of experts, including surgeons and gastroenterologists, retrospectively assessed changes in CDM. Each expert individually reviewed cases and rated whether NGS influenced CDM. Disagreements were resolved via the Delphi technique 28 .

A change in CDM due to NGS outcome was defined as alteration in the treatment course or a decisive factor to continue the current treatment, without requiring additional imaging or pathology material.

No change in CDM due to NGS outcome was defined as: 1) Cases with suspicious pathology or high suspicion on imaging received surgical treatment regardless of NGS outcome; 2) If both pathology and imaging were suspicious for malignancy chemotherapy was given, without needing a positive NGS outcome; 3) Best supportive care was provided for patients unfit for treatment or desired no treatment; 4) No targeted therapy options were available based on the mutations shown by NGS; 5) Unsuccessful NGS; and 6) In cases with low suspicion of malignancy, a negative NGS outcome did not influence treatment or additional follow-up with imaging and repeated pathology was needed to conform benign pathology.

### Data collection

The medical records were systematically reviewed and data were collected pseudo-anonymously on patient demographics (age, gender, history of primary sclerosing cholangitis (PSC)), tumor location, tumor markers, radiological findings, bile duct characteristics such as number of biopsies/brushes, location and pathological outcome, NGS panel used, NGS outcome, and mutations found. Information about clinical follow-up regarding treatment, diagnostics, and survival was retrieved.

### Statistical analysis

The statistical analysis contained descriptive statistics using medians (with interquartile ranges [IQRs]) for not normally distributed continuous variables and using frequencies and proportions for categorical and dichotomous variables. Sensitivity, specificity, and accuracy were calculated using standard 2 × 2 contingency tables without repeated testing for individual biomarkers. Statistical analyses were performed with SPSS Statistical software version 28, with significance defined as *P* < 0.05.

## Results

### Baseline characteristics

During the study period a total of 691 samples were obtained from 444 patients. We initially identified 113 patients, of whom four were excluded based on exclusion criteria. Three patients had insufficient follow-up and were also excluded, resulting in 106 patients for analysis. Table 1 shows baseline characteristics. Clinical and diagnostic pathology follow-up showed 75 malignant and 31 benign cases.

**Table:** **Table 1** Characteristics of patients with biliary strictures.

	Total (n = 106)				
**Female gender, n (%)**	50	(47.2)			
**Age at diagnosis in years, median (IQR)**	67	(58–75)			
**History of PSC, n (%)**	18	(17.0)			
CA-19.9 in kU/L, median (IQR)*	183	(50–629)			
**Bilirubin level in µmol/L, median (IQR)**	54	(12–183)			
**Location of stricture, n (%)**					
Intrahepatic	8	(7.5)			
Hilar	55	(51.9)			
Distal	43	(40.6)			
**Clinical and diagnostic pathology follow-up**	**Total**	**Resection**	**Biopsy**	**Imaging**	**Clinical**
Perihilar cholangiocarcinoma	43	17	7	11	8
Pancreatic ductal adenocarcinoma	13	7	3	2	1
Distal cholangiocarcinoma	10	2	4	4	
Intrahepatic cholangiocarcinoma	5	2	1	1	1
Ampullary adenocarcinoma	2	2			
Hepatocellular carcinoma	1		1		
Duodenal carcinoma	1	1			
Benign cholangiopathy	31				
PSC	13	2		11	
Low grade biliary dysplasia	1	1			
Other	17	3		14	
*Serum CA-19.9 was measured for 91 of 109 patients (83%). CA-19.9, carbohydrate antigen 19.9; IQR, interquartile range; PSC, primary sclerosing cholangitis.					

Table 2 outlines characteristics of brushes, biopsies, and NGS panels. In total 109 samples were obtained from 106 patients, including 94 brushes (86%) and 15 biopsies (14%). Three biopsies were cholangioscopy guided. In 10 cases, the initial NGS analysis was extended with an additional panel. The extended analysis showed a change from negative to positive in five cases (50%).

**Table:** **Table 2** Characteristics of brushes, biopsies, and next-generation sequencing.

	** Total ** n = 109		
**Brushes, n (%)**	94 (86.2)		
**Biopsies, n (%)**	15 (13.8)		
**Academic center, n (%)**	91 (83.5)		
**Technique, n (%)**			
ERCP	104 (95.4)		
PTBD	5 (4.6)		
**Initial NGS panel, n (%)**			
Pan-cancer panel v5.1	8 (7.3)		
Pan-cancer panel v6.1	23 (21.1)		
CfDNA	66 (60.6)		
Unsuccessful	12 (11.0)		
**Initial panel to extended NGS panel**		**Result NGS**	**Clinical FU**
Pan-cancer V5.1 to cfDNA	1	Both positive	Malignant
Pan-cancer V6.1 to cfDNA	1	Both negative	Malignant
cfDNA to Pan-cancer V5.1	1	Both positive	Malignant
cfDNA to Pan-cancer V6.1	6	5x Initially negative → positive	Malignant
		1x both negative	Benign
cfDNA to unsuccessful	1	Negative	Malignant
cfDNA, cell-free DNA; ERCP, endoscopic retrograde cholangiopancreatography; FU, follow-up; NGS, next-generation sequencing; PTBD, percutaneous transhepatic biliary drainage.			

### Morphology outcome

Morphology alone correctly identified 43 of 75 malignancies (57%). Morphology showed three false positives and 32 false negatives ( Table 3 ). False positives had all been classified as suspicious for malignancy (3/40). Overall sensitivity of morphology alone in the brushes and biopsies was 56% (95% confidence interval [CI] 43%-68%) and 67% (95% CI 35%-90%) and specificity 94% (95% CI 79%-99%) and 67% (95% CI 9%-99%), respectively. Brushes and biopsies showed a PPV of 95% (95% CI 82%-99%) and 89% (95% CI 61%-98%) and NPV of 51% (95% CI 44%-58%) and 33% (95% CI 14%-61%), respectively.

**Table:** **Table 3** Correlation of morphologic diagnosis, NGS outcome, and clinical follow-up, with outcome of extended panel included.

	Morphologic diagnosis	NGS outcome	Clinical follow-up		Total
			Benign	Malignant	
Brush	Benign (n = 5)	Negative	3		**3**
		Unsuccessful		2	**2**
	Atypical (n = 52)	Negative	23	8	**31**
		Positive		14	**14**
		Unsuccessful	3	4	**7**
	Suspicious for malignancy (n = 31)	Negative	2	9	**11**
		Positive		18	**18**
		Unsuccessful		2	**2**
	Malignant (n = 6)	Negative		2	**2**
		Positive		3	**3**
		Unsuccessful		1	**1**
Biopsy	Atypical (n = 6)	Negative	2	4	**6**
	Suspicious for malignancy (n = 9)	Negative	1	1	**2**
		Positive	0	7	**7**
Total			34	75	109*
* Three patients received a brush and biopsy with NGS analyses. NGS, next-generation sequencing.					

### NGS outcome

NGS showed positive results in 42 cases and negative results in 55 cases, including the outcome of the 10 cases with an extended panel. NGS was unsuccessful in 12 cases NGS due to insufficient material ( Table 3 ). NGS correctly identified 42 of 75 malignancies (56%), with no false positives and 24 false negatives. Overall sensitivity and specificity of NGS analyses on brushes and biopsies showed a sensitivity of 56% (95% CI 42%-68%) and 58% (95% CI 27%-85%), with both 100% (95% CI 89%-100%) and 100% (95% CI 29%-100%) specificity, respectively. Especially for patients with indecisive atypical morphology (n = 58), NGS correctly classified 14 of 30 malignancies (47%). NGS on brushes and biopsies showed both a PPV of 100% and a NPV of 53% (95% CI 46%-59%) and 38% (95% CI 24%-54%), respectively. Sensitivity, specificity, accuracy, PPV, and NPV of NGS for each morphologic category are shown in Table 4 .

**Table:** **Table 4** Sensitivities, specificities, accuracy, PPV, and NPV of NGS divided per morphological outcome.

	Sensitivity NGS [95% CI]	Specificity NGS [95% CI]	Accuracy NGS [95% CI]	PPV NGS [95% CI]	NPV NGS [95% CI]
Brush benign (n = 5)	0.00 [0.00–0.84]	1.00 [0.29–1.00]	0.60 [0.15–0.95]	N.A.	0.60 [0.60 – 0.60]
Brush atypical (n = 52)	0.54 [0.33–0.73]	1.00 [0.87–1.00]	0.77 [0.63–0.87]	1.00	0.68 [0.59–0.77]
Brush suspicious for malignancy (n = 31)	0.62 [0.42–0.79]	1.00 [0.16–1.00]	0.65 [0.45–0.81]	1.00	0.15 [0.10–0.22]
Brush malignant (n = 6)	0.50 [0.12–0.88]	N.A.	N.A.	1.00	0
** All brushes (n = 94) **	0.56 [0.42–0.68]	1.00 [0.89–1.00]	0.70 [0.60–0.79]	1.00	0.53 [0.46–0.59]
Biopsies atypical (n = 6)	0.00 [0.00–0.60]	1.00 [0.16–1.00]	0.33 [0.04–0.78]	N.A.	0.33 [0.33–0.33]
Biopsies suspicious for malignancy (n = 9)	0.88 [0.47–0.99]	1.00 [0.03–1.00]	0.89 [0.52–1.00]	1.00	0.50 [0.14–0.86]
** All biopsies (n = 15) **	0.58 [0.27–0.85]	1.00 [0.29–1.00]	0.66 [0.38–0.88]	1.00	0.38 [0.24–0.54]
CI confidence interval; NGS, next-generation sequencing; NPV, negative predictive value; PPV, positive predictive value.					

In a subgroup analysis of 17 patients with PSC, NGS on brushes showed a sensitivity of 75% (95% CI 19%-99%), specificity of 100% (95% CI 75%-100%), and accuracy of 93% (95% CI 71%-100%).

### Morphology combined with NGS

Combining NGS results with morphology, a sensitivity of 78% (95% CI 66%-87%), specificity of 94% (95% CI 79%-99%), accuracy of 83% (95% CI 74%-90%), PPV of 96% (95% CI 86%-99%), and NPV 67% (95% CI %56–77%) was reached for all brushes and a sensitivity of 67% (95% CI 35%-90%), specificity of 67% (95% CI 9%-99%), accuracy of 67% (95% CI 38%-88%), PPV of 89% (95% CI 61%-98%) and NPV 33% (95% CI 14%-61%) for all biopsies. Results excluding the unsuccessful NGS outcome are presented in **Supplementary Text 2** . **Supplementary Table 1** shows results of the initial NGS analyses.

### Mutations identified by NGS

**Fig. 1:**
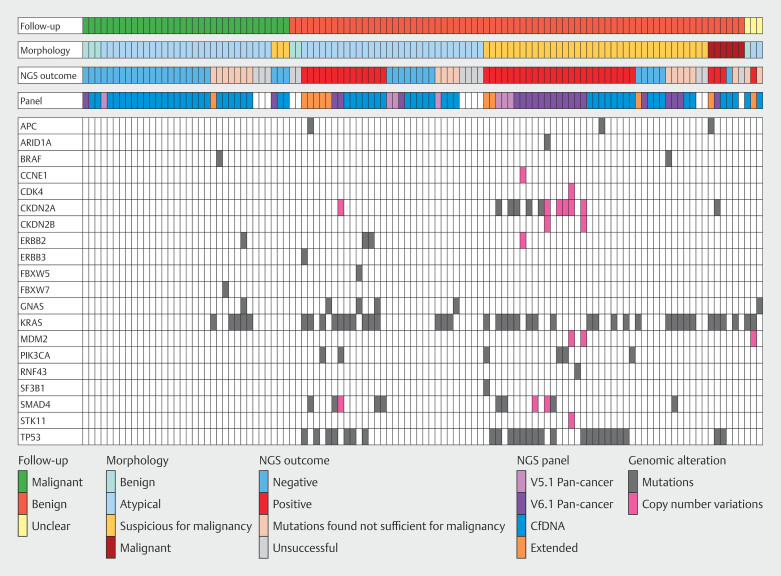
Correlation of follow-up, morphological outcome and genomic alterations.

**Fig. 2:**
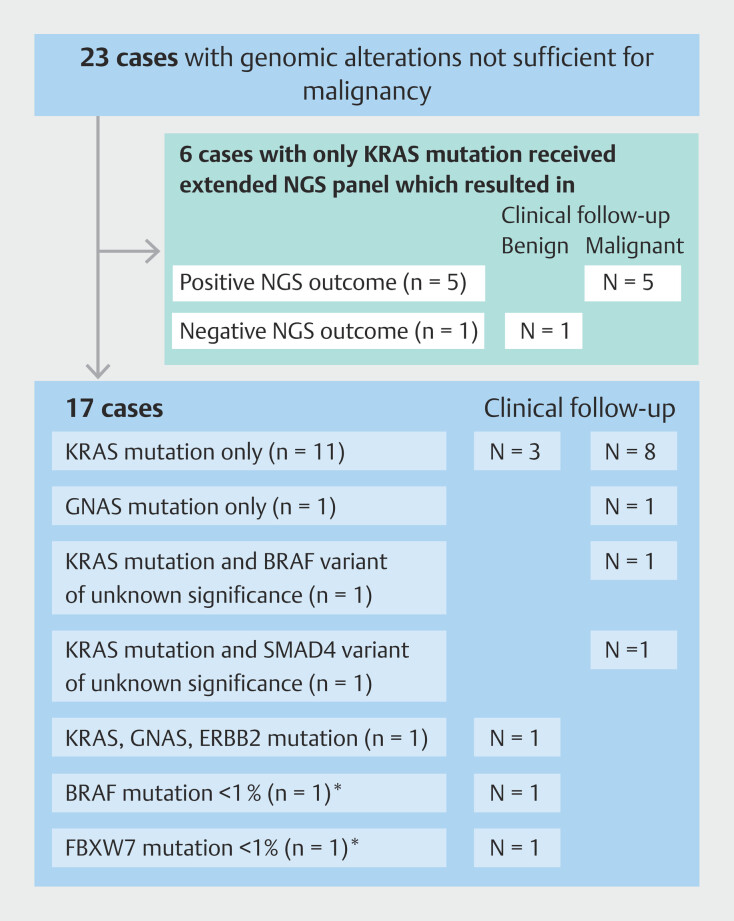
Genomic mutations classified “not sufficient for malignancy” with follow-up in benign and malignant pathology. * These mutations were present in < 1% of genes and, therefore, classified as insufficient for a positive NGS result.

Fifteen genes with mutations were found, most often in *KRAS* (n = 46), *TP53* (n = 28), and *SMAD4* (N =8) ( Fig. 1 ). Of the 23 cases with genomic alterations that were deemed insufficient to qualify as a positive NGS result, 13 cases showed a solitary *KRAS* mutation with three of 13 (23%) benign disease and 10 of 13 (77%) malignant disease at follow-up ( Fig. 2 ). Two of three patients with a *KRAS* mutation and benign follow-up were diagnosed with PSC. Reclassifying these genomic alterations as positive NGS outcomes would increase sensitivity to 73% (95% CI 60%-83%) but decrease specificity to 81% (95% CI 63%-93%). Fig. 1 provides an overview of follow-up results related to morphologic outcome, NGS result, NGS panel and various mutations, deletions, and amplifications.

### Clinical decision-making after NGS

In nine of 106 patients (8%), NGS caused a change in CDM. In two cases, atypical brushings with non-suspicious imaging a positive NGS resulted in surgery, revealing malignancy upon resection. In two cases with atypical brushings, positive NGS allowed chemotherapy without additional pathology proof. One patient for whom surgery initially was recommended due to highly suspicious imaging and an atypical brush result opted for surgery only after a more certain diagnosis was confirmed by a positive NGS. A negative NGS outcome in one case led to continued follow-up instead of the initial recommended surgery, with subsequent benign disease. Finally, in three patients, NGS was able to distinguish the origin of a metastatic lesion in patients with both colon carcinoma and CCA, leading to a different chemotherapy regimen. **Supplementary Text 3** and Fig. 1 elaborate on the 97 cases in which NGS did not alter CDM.

### NGS impact on pathological classification

**Fig. 3:**
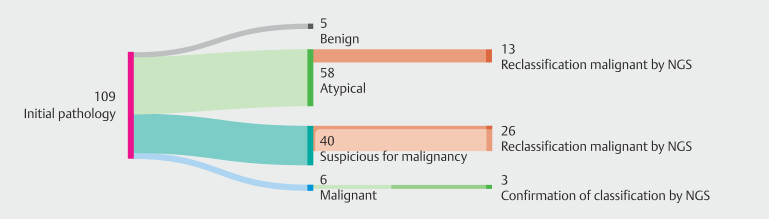
Change of pathology classification due to positive NGS outcome.

A positive NGS outcome resulted in reclassification of 39 of 109 samples (36%) from their original pathology classification. Thirteen atypical classifications and 26 suspicious for malignancy classifications were changed to malignant based on a positive NGS outcome. In addition, three malignant classifications were supported by a positive NGS result, for one of which a distinction in primary tumor could be made. A graphic overview is shown in Fig. 3 .

## Discussion

Accurate diagnostic methods for classifying suspicious biliary stricture in benign and malignant lesions are still lacking. We demonstrated in a cohort of 106 patients that adding NGS data to morphology increases sensitivity of brushes. Our study contributes to current literature by examining a substantial number of brushes with indecisive atypical morphology. In these brushes, NGS correctly identified 14 of 30 malignant cases. Importantly, NGS influenced CDM in 8% of the cases, allowing correct and earlier treatment for a selection of patients.

Several studies have been published on the sensitivity and specificity of NGS alone in suspicious biliary strictures. Dudley et al. and Harbhajanka et al. reported a sensitivity of NGS on biliary brushes of 73% and 93% and a specificity of 98% and 100%, respectively 24 25 . Singhi et al. also included intraductal biopsies and report a sensitivity of 74% and a specificity of 100% 23 . Two recent studies showed sensitivities of 75% for brushes and biopsies and 85% for brushes 29 30 . Overall, these data demonstrate a relatively high overall sensitivity and specificity of NGS on biliary specimens. The differences in sensitivity between these studies and our study might be attributed to case mix, variations in NGS panel sizes, and detection limits. For example, the cfDNA panel has a much higher detection limit than our pan-cancer panels (1% vs 10%), but analyzes only a limited number of genes. The previously mentioned studies reported a lower detection limit of 3% to 5% as well as broader gene panels. In addition, differences in sensitivity may result from excluding unsuccessful NGS results, which is not clearly described in the methods of these articles. Including the unsuccessful NGS results is a strength of our study, because it reflects clinical practice. Furthermore, Harbhajanka et al. demonstrated a significantly higher sensitivity (93%), which was probably largely influenced by a considerable number of patients with benign morphology in the initial brush, reducing the likelihood of false-negative NGS outcomes 22 .

In addition, in contrast to the above-mentioned studies, we did not classify all pathologic mutations as positive NGS outcomes. Specifically, single mutations in *KRAS* or *GNAS* were deemed "not sufficient for malignancy" and classified as negative outcome. All other studies diagnosed a specimen as malignant when any pathologic mutation in a known tumor-associated gene was present, even if only *KRAS* or *GNAS* was mutated. Single mutations in *KRAS* or *GNAS* are known to have lower specificity for malignancy compared with late pathway mutations such as *TP53* , *SMAD4* , or *CDKN2A* 31 . If we classify “not sufficient for malignancy” mutations as positive NGS result, sensitivity increases to 85%, but specificity decreases to 79%. This aligns with Rosenbaum et al., who found that including single mutations in *KRAS* or *GNAS* increases sensitivity on brushes to 100%, but dramatically lowers specificity from 100% to 73% 32 . Especially in PSC, it is known that *KRAS* mutations do not always result in CCA, although they are often involved in early development of CCA 33 . Like our study, Kamp et al. reported two patients with benign PSC disease and single *KRAS* and *GNAS* mutations 34 . Therefore, classifying solitary *KRAS* and *GNAS* mutations as a positive result for malignancy should be done with caution. Particularly for patients with PSC, there is a risk of unnecessary treatment with chemotherapy or surgery due to a false-positive NGS result.

Our study showed a false-negative NGS result in 24 cases (32%), for several reasons. First, sampling error during biliary duct brushes or intraductal biopsy can result in non-representative material. The success of NGS analysis relies on the quality, quantity, and representativity of the sample. Diagnostic yield may be improved by incorporating cholangioscopy-guided biopsies, because they enable more precise tissue sampling. Nevertheless, Singhi et al. found no statistically significant differences in sensitivity and specificity between cholangioscopy-guided and fluoroscopy-guided biopsies in 28 samples 23 . This may be attributable to the limited sample size, and future studies with statistical power may demonstrate the benefit of combining cholangioscopy with NGS. Second, our panels are not specifically designed for CCA, but are intended for pancreatic and colon carcinoma. Mirallas et al. provided an overview of the most common mutations found in intrahepatic and extrahepatic CCA by whole genome sequencing 35 . The most common mutations in *KRAS* , *TP53* , *SMAD4* , and *CDKN2A* are detected by our panels, but those in *ARID1A* , *BAP1* , *IDH1* , and *CDKN2A* were not included in the cfDNA panel. Knowledge about genomic alterations in CCA will help to develop a more tailored and sensitive NGS panel designed for CCA.

NGS results changed CDM in 8% of cases. This rate is partly due to the high clinical suspicion of malignancy based on imaging and tumor markers, such as CA-19.9. Regardless of the outcome of NGS or even the morphologic outcome, treatment was often continued as planned. In addition, a negative NGS result does not completely rule out malignancy. Additional radiological and pathological diagnostics were often repeated in order to determine benign pathology. Nevertheless, in almost one of 10 cases, a positive NGS result led to faster and better management for patients. A recent study underscored the positive impact on management changes due to a positive NGS result 30 .

In contrast to morphologic examination, NGS analysis is relatively expensive and this may increase with use of larger panels. For example, compared with cholangioscopy, NGS is often more expensive. Nevertheless, we believe that NGS could be cost-effective for indeterminate biliary strictures because it can be performed on previously obtained samples, whereas cholangioscopy, as currently recommended in clinical guidelines, requires a new procedure with associated risks of complications 14 . Furthermore, NGS had demonstrated a higher sensitivity compared with cholangioscopy. To optimize use of resources, consideration could be given to selectively performing NGS only in cases in which the result may lead to changes in CDM. Based on our findings we would recommend performing NGS in two scenarios. The first is in all patients with indecisive atypical morphology. NGS clarified malignant diagnosis in 14 of 58 of our patients, avoiding additional expensive diagnostics and unnecessary follow-up. The second scenario is in patients with two primary tumors, such as patients with colon carcinoma as described in the results in which NGS can differentiate the origin of metastatic lesions. In the future, NGS will have a greater impact on CDM, as targeted treatment options for CCA and pancreatic carcinoma expand, including US Food and Drug Administration-approved options for mutations in the *FGFR* pathway, *IDH1* , *ERBB2* , *BRCA1/2* , and microsatellite-instable tumors. Ongoing research is exploring treatments for *BRAF* , *ALK* , and *MET* mutations, with successful targeted chemotherapy for *HER2* amplification identified by NGS 36 .

Although in many countries targeted therapies are still unavailable or limited, recent European Society for Medical Oncology guidelines recommend NGS for patients with advanced cancers in centers where these targeted therapies are accessible 37 .

In this article, we primarily focused on the clinical impact of the NGS outcome. However, NGS outcomes also have a significant impact on reclassifying pathology because they provide greater certainty about malignancy. The results showed that in 36% of the samples, a definite diagnosis of malignancy could be given by the pathologist instead of an uncertain result. In comparison, Harbhajanka et al. showed an almost similar reclassification of pathology due to NGS with 40 of 94 samples (43%) 25 .

Our study contributes to current literature because it included a large number of patients with atypical morphology in brushes, where CDM is especially difficult. In addition, this study evaluated not only NGS test outcomes such as sensitivity and specificity similar to other studies, but it also systematically assessed clinical impact of NGS. As with most retrospective studies, this study has some limitations. First, different NGS panels were used depending on the estimated percentage of neoplastic cells, with both panels having their own limitations, as mentioned previously. With the extended panel, we reduced this problem by searching for additional mutations. Second, NGS analysis of the biopsies was underpowered due to small numbers, requiring a larger cohort to provide a stronger statement about its effectiveness. Third, there likely was a difference in NGS results in patients with benign versus atypical or suspicious for malignancy versus malignant morphology. For calculation of sensitivity and specificity, we combined these results, in line with other articles on this subject 23 24 25 . Finally, the retrospective design poses a risk of selection bias. There were no clear guidelines for requesting NGS by clinicians. Clinicians may have requested NGS only for highly suspicious strictures, which could have led to an incorrect assumption. In addition, the determination of CDM change caused by NGS was retrospective. Although that is prone to bias, by discussing the cases with a group of experts, the influence of NGS on CDM was weighed as thoroughly as possible. Despite these limitations, our study highlights the contribution of NGS in optimizing diagnostics of biliary strictures and CDM.

## Conclusions

In conclusion, NGS on biliary duct brushes adds value to morphology alone in diagnosing malignant biliary strictures. Especially in indecisive atypical morphology, NGS is able to detect malignancies and consequently reduces treatment delay. Our study showed a limited change in CDM, but in the future, NGS will undoubtedly play a bigger role when targeted therapy is incorporated in standard treatment and more sensitive NGS panels for cholangiocarcinoma are developed.

## Supplementary Material

Supplementary Material Zusatzmaterial
