# Supplementary material for: Yield of next-generation sequencing in diagnostic work up of suspicious biliary strictures
Source: Endosc Int Open. 2025 Sep 5;13:a26873552. doi: 10.1055/a-2687-3552 (PMC12417792; doi:10.1055/a-2687-3552)
Supplement: Supplementary file 1 — Supplementary Material [file 10-1055-a-2687-3552_26896314.pdf]

Supplementary Text 1 Exons analyzed per gene in different NGS panels.

NGS Pan-cancer panel V5.1:

Entire coding regions: *CDKN2A* (98% coverage), *PTEN* (94%), *TP53* (100%).

Mutation hotspots: *AKT1* (exon 3), *ALK* (20, 22-25), *APC* (14), *ARAF* (7), *BRAF* (11, 15), *CTNNB1* (3, 7, 8), *EGFR* (18-21), HER2 (19-21), *EZH2* (16), *FBWX7* (9, 10), *FGFR1* (4, 7, 12), *FGFR2* (7, 9, 12), *FGFR3* (7, 9), *FOXL2* (1), *GNA11* (4, 5), *GNAQ* (4, 5), *GNAS* (8, 9), *HRAS* (2-4), *IDH1* (4), *IDH2* (4), *KIT* (8, 9, 11, 13, 14, 17), *KRAS* (2-4), *MAP2K1* (2, 3), *MET* (2, 14, 19), *MYD88* (5), *NOTCH1* (26, 27), *NRAS* (2-4), *PDGFRA* (12, 14, 18), *PIK3CA* (10, 21), *POLD1* (12), *POLE* (9, 13), *RAF1* (7), *RET* (11, 16), *RNF43* (3, 4, 9), *ROS1* (38, 41), *SMAD4* (3, 9, 12), *STK11* (4, 5, 8).

Non-coding region: TERT promoter.

NGS Pan-cancer panel V6.1:

Entire coding regions: *CDKN2A* (100% coverage), *KEAP1* (100%), *PTEN* (100%), *STK11* (100%), *TP53* (100%).

Mutation hotspots: *AKT1* (exon 3), *AKT2* (3), *AKT3* (2), *ALK* (20, 22-25), *APC* (16), *ARAF* (7), *BRAF* (11, 12, 14, 15), *CDK4* (2, 4, 7, 8), *CHEK2* (3, 4, 11, 12), *CTNNB1* (3, 7, 8), *DDR2* (14-19), *EGFR* (12, 18-21), *EIF1AX* (1, 2), HER2 (8, 17-21), *ERBB3* (3, 6-10, 21, 23), *ESR1* (4, 5, 7, 8), *EZH2* (16), *FBWX7* (9, 10), *FGFR1* (4, 7, 12-14), *FGFR2* (7, 9, 12), *FGFR3* (7, 9, 14, 15), *FOXL2* (1), *GNA11* (4, 5), *GNAQ* (4, 5), *GNAS* (8, 9), *HRAS* (2-4), *IDH1* (4), *IDH2* (4), *JAK2* (14), *JAK3* (4, 16), *KIT* (8, 9, 11, 13-18), *KNSTRN* (1), *KRAS* (2-4), *MAP2K1* (1-6), *MET* (2, 14, 19, 20), *MTOR* (30, 39, 40, 43,

Endosc Int Open | DOI 10.1055/a-2687-3552 | © The Author(s).

47, 53, 56, 57), *MYD88* (5), *NFE2L2* (2), *NOTCH1* (26, 27), *NRAS* (2-4), *OXA1L* (1), *PDGFRA* (12, 14, 18), *PIK3CA* (2, 5, 8, 10, 14, 21), *POLD1* (6, 8, 12, 15-17, 24), *POLE* (9-14, 21, 25), *RAC1* (2), *RAF1* (7), *RET* (11, 16), *RHOA* (2), *RIT1* (4, 5), *RNF43* (2-10), *ROS1* (36-41), *SF3B1* (14, 15), *SMAD4* (3, 9, 12).

Non-coding region: TERT promoter.

Oncomine Colon cell free DNA (cfDNA) Assay V1:

Fourteen genes with >240 hotspots covered: *AKT1*, *APC*, *BRAF*, *CTNNB1*, *EGFR*, *ERBB2*, *FBXW7*, *GNAS*, *KRAS*, *MAP2K1*, *NRAS*, *PIK3CA*, *SMAD4*, *TP53*.

Endosc Int Open | DOI 10.1055/a-2687-3552 | © The Author(s).

Supplementary Text 2 Calculation with the unsuccessful NGS brushes included in all categories.

Morphology alone showed a sensitivity, specificity, PPV, and NPV of 56%, 94%, 95%, and 51%, respectively. NGS showed a sensitivity, specificity, accuracy, PPV, and NPV of NGS as follows: benign (N.A%, 100%, N.A., N.A, 100%), atypical (64%, 100%, 82%, 100%, 74%), suspicious for malignancy (67%, 100%, 69%, 100%, 18%), malignancy (60%, N.A., N.A., 100%, 0%) and all brushes (65%, 100%, 76%, 100%, 60%). When the NGS results are combined with morphology, a sensitivity of 85%, specificity of 93%, accuracy of 88%, PPV of 96%, NPV of 78% is reached for all brushes.

Endosc Int Open | DOI 10.1055/a-2687-3552 | © The Author(s).

Supplementary Text 3 Explanation of patients where CDM was not altered.

In 97 patients NGS did not influence clinical decision-making (CDM). **Supplementary Fig. 1** provides a schematic overview of these patients. The patients were divided in two morphological groups benign/atypical (n = 60) and suspicious for malignancy/malignant (n = 46), because the treatment choice primarily depends on the morphological outcome. For every patient the following was noted: 1) if they had a high suspicion for malignancy on imaging or not; 2) if they had a positive or negative NGS result; and 3) which final treatment was given to the patient. The different imaging used to determine suspicion for malignancy were computed tomography (CT) scan, magnetic resonance imaging (MRI), and endoscopic retrograde cholangiopancreatography (ERCP).

The patients can be divided in the following subgroups:

- If there was a high suspicion of malignancy on imaging, patients received surgical treatment regardless of a negative or positive NGS outcome (benign/atypical n = 14, at least suspicious for malignancy n = 14).
- Patients received surgical treatment based on suspicious morphology alone. When no clear suspicion of malignancy on imaging was seen and regardless of the NGS outcome, surgical treatment was performed as planned (n = 5). However, there was one exception: One patient had no suspicion of malignancy on imaging, a negative NGS outcome, but suspicious morphology and did not receive surgical treatment. Imaging of this patient showed many more signs of inflammation than of malignancy, which resulted in additional pathology and imaging, which did not show any sign of malignancy.

Endosc Int Open | DOI 10.1055/a-2687-3552 | © The Author(s).

- When patients had a combination of suspicious morphology and a high clinical suspicion of malignancy on imaging, they received chemotherapy regardless of the NGS outcome (n = 10).
- In patients with atypical morphology and high suspicion on imaging, additional pathology proof for chemotherapy treatment was indicated, because the NGS outcome was negative (n = 4). Two patients with atypical morphology had initially high suspicion of malignancy on imaging and a negative NGS result. For chemotherapy treatment, pathological proof was indicated. Eventually multiple brushes and biopsies did not show malignancy and imaging showed involution or no progression of the lesion and the cases were classified benign.
- Two patients with pancreatic head carcinoma were screened for targeted therapy. The mutations *KRAS*, *GNAS*, and *SMAD4* in one patient and a *KRAS* mutation in the other patient were not suitable for targeted therapy.
- Patients received best supportive care, because they were unfit for treatment or wished no treatment (n = 14).
- Some patients had a very low clinical suspicion of malignancy, for example, a biliary stricture after a liver transplantation in a primary sclerosing cholangitis patient. Imaging also did not show any signs of malignancy. A negative NGS outcome was not necessary and follow-up was not indicated (benign/atypical n = 4).
- In other cases, a negative NGS outcome and no suspicion on imaging was not enough to confirm benign pathology. Follow-up with CT, MRI, ERCP

Endosc Int Open | DOI 10.1055/a-2687-3552 | © The Author(s).

(benign/atypical n = 8), or repeated pathology (benign/ atypical n = 7) was deemed necessary to confirm the benign pathology.

- Lastly, there were cases in which NGS was unsuccessful and could not change CDM (n = 12).

Endosc Int Open | DOI 10.1055/a-2687-3552 | © The Author(s).

Supplementary Table 1 NGS outcome without results of extended panel.

	Morphologic diagnosis	NGS outcome	Total	Clinical follow-up	
				Benign	Malignant
Brush	Benign	Negative	3	3	0
	n = 5	Positive	0	0	0
		Unsuccessful	2	0	2
	Atypical	Negative	34	23	11
	n = 52	Positive	11	0	11
		Unsuccessful	7	3	4
	Suspicious for malignancy	Negative	13	2	11
	n = 31	Positive	16	0	16
		Unsuccessful	2	0	2
	Malignant	Negative	3	0	3
	n = 6	Positive	2	0	2
		Unsuccessful	1	0	1
Biopsy	Atypical	Negative	6	2	4
	n = 6	Positive	0	0	0
		Unsuccessful	0	0	0
	Suspicious for malignancy	Negative	2	1	1
	n = 9	Positive	7	0	7
		Unsuccessful	0	0	0
Total			109*	34	75

*In three patients, a brush and biopsy were analyzed by NGS.

Endosc Int Open | DOI 10.1055/a-2687-3552 | © The Author(s).

Supplementary Figure 1 Explanation of patients without influence of Next Generation Sequencing on clinical decision-making. Received treatment is showed divided in morphological outcome benign/atypical and at least suspicious for malignancy, (no) suspicion on imaging for malignancy and NGS outcome.
* Pathology proven before given chemotherapy; Imaging +/- = imaging suspicious/ not suspicious for malignancy; NGS +/- = positive/negative NGS result; CDM +/- = change or no change in CDM; CDM= Clinical descision-making; NGS= Next generation sequencing; BSC= Best supportive care; FU= Follow-up, Re PA= Pathology (brush or biopsy) repeated.

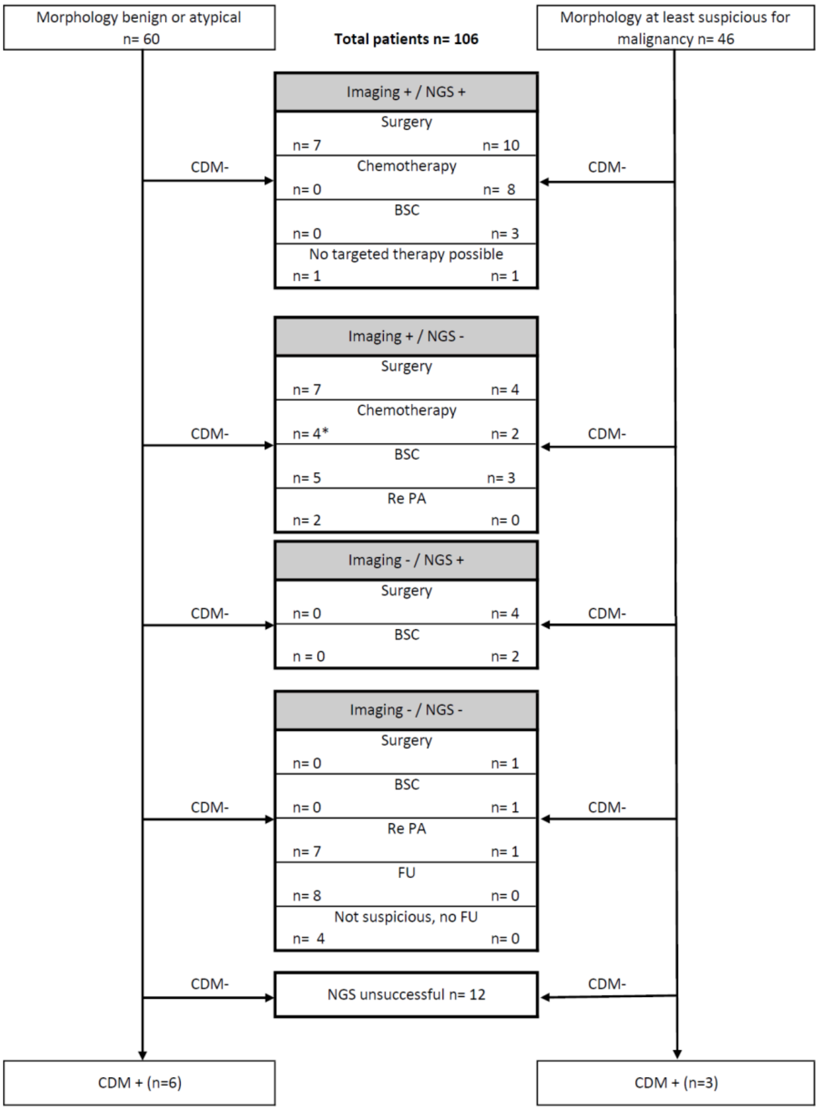

Endosc Int Open | DOI 10.1055/a-2687-3552 | © The Author(s).
